# Basal Cell Carcinoma Associated with Secondary Localized Cutaneous Amyloid Deposits: Case Report and Review

**DOI:** 10.7759/cureus.3879

**Published:** 2019-01-14

**Authors:** Philip R Cohen

**Affiliations:** 1 Dermatology, San Diego Family Dermatology, San Diego, USA

**Keywords:** amyloid, amyloidosis, basal, carcinoma, cell, cutaneous, cytokeratin, deposits, localized, secondary

## Abstract

Amyloid deposition has been observed in tissue specimens of basal cell carcinomas. A 68-year-old man with a nodular basal cell carcinoma on his left arm near the elbow is described; microscopic evaluation of the biopsy tissue specimen shows not only nodular aggregates and strands of atypical basaloid tumor cells but also marked deposition of amorphous amyloid material in the stroma between the aggregates of basal cell carcinoma. Including the man in this report, there are additional individual descriptions of patients whose basal cell carcinomas have amyloid deposits in the adjacent stroma or within the tumor aggregates or both. In addition, several retrospective pathology investigations have evaluated the features of this phenomenon. The incidence of basal cell carcinoma with amyloid deposition, in the English literature, ranges from 11% to 75%; however, it is possible that staining technique or tumor subtype or quantity of amyloid present may account for the lower detection of amyloid observed by some of the researchers. Amyloid in basal cell carcinoma specimens was observed to be present more frequently in older patients who had tumors with less aggressive histology patterns. Nodular basal cell carcinoma was the most common subtype of tumor with amyloid deposits whereas superficial basal cell carcinoma was the least frequent subtype. The amyloid deposits were usually identified on hematoxylin and eosin-stained sections and confirmed by using stains that allowed for easier visualization of the amyloid. The amyloid deposits were most commonly located in the stroma between the tumor aggregates; other locations included the papillary dermis above the carcinoma, the dermis at the advancing edge of the tumor and within the aggregates of basal cell carcinoma. Many of the basal cell carcinomas with amyloid deposits, similar to the reported patient, also contained solar elastosis. The origin of the amyloid deposition in these tumors is secondary amyloid AA protein from keratin derived from the epithelial cells overlying the basal cell carcinomas. The presence of amyloid deposition does not alter the management of these basal cell carcinomas; the treatment of the tumor is the same as when the basal cell carcinoma does not contain amyloid deposition.

## Introduction

The presence of exogenous material (such as bone) or infection (such as *Mycobacteria leprae*) within the tumor nodules or the adjacent dermal stroma or both has been observed in cutaneous specimens of basal cell carcinoma [1-2]. Basal cell carcinoma with amyloid deposition has also been described—more commonly in retrospective studies [3-13] and less frequently in case reports [14-17]. A man with a basal cell carcinoma that had localized amyloid deposits is described and the features of secondary amyloid deposition in cutaneous basal cell carcinoma are reviewed.

## Case presentation

A 68-year-old man presented for evaluation of a new asymptomatic bump on his left arm. His previous skin examination had been six months earlier and the lesion had not been present. He previously had three basal cell carcinomas (on the left temple, left side of his upper lip, and left mid back excised 32 years, four years and two years earlier, respectively) and one squamous cell carcinoma (on his right upper back that was excised seven years ago). He also had actinic keratoses that were treated with liquid nitrogen cryotherapy.

His past medical history was significant for severe acne vulgaris as an adolescent, hypercholesterolemia, hypertension, and prostate cancer that was diagnosed one year ago. He is currently with no evidence of malignant disease after treatment which included a robotic-assisted laparoscopic prostatectomy (with negative margins for tumor) and a bilateral pelvic lymph node dissection (with none of eight nodes positive for cancer). His current oral daily medications included amlodipine 10 mg and simvastatin 20 mg.

**Figure 1:**
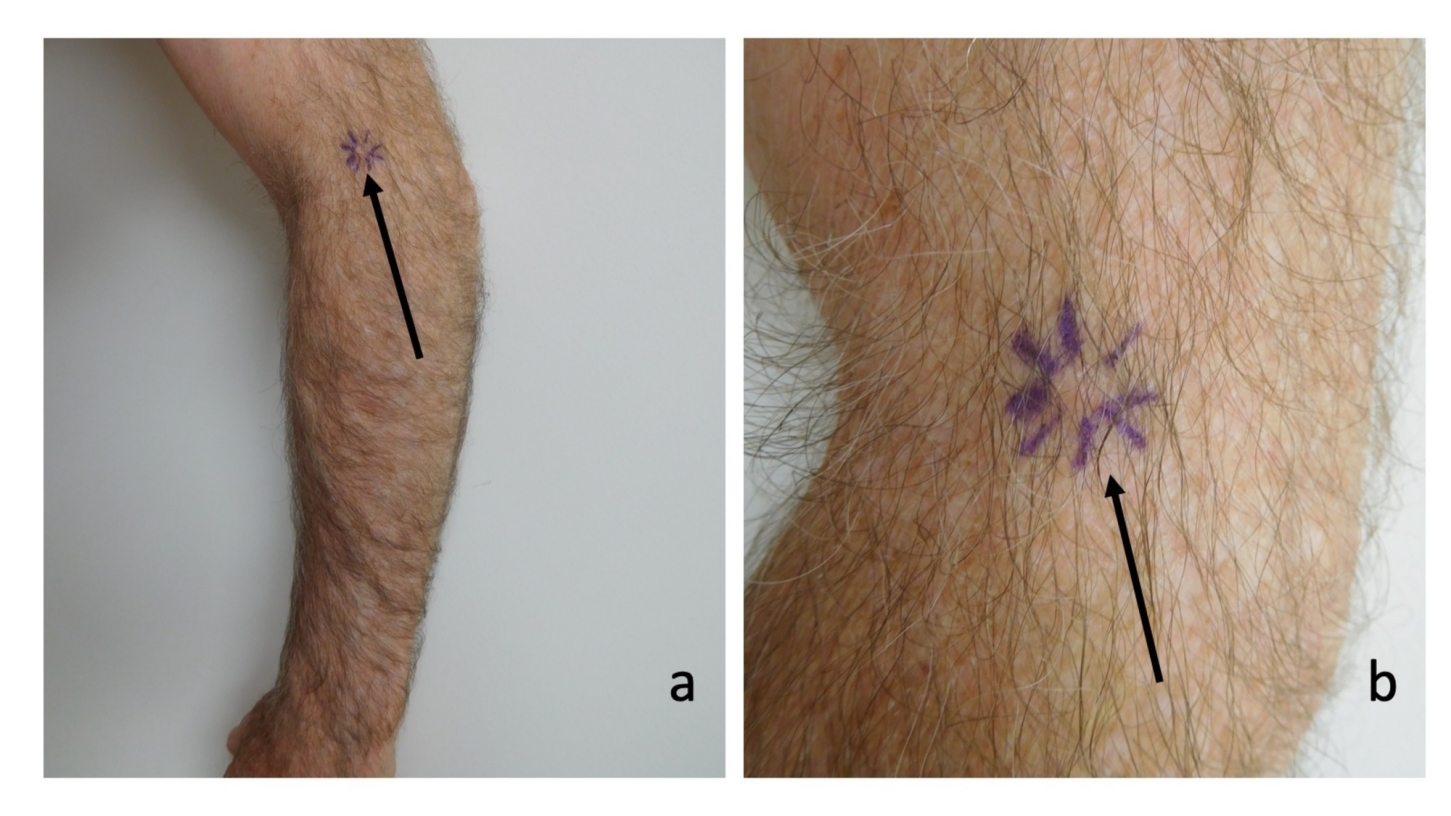
Basal cell carcinoma with amyloid deposits on the left arm. Distant (a) and closer (b) views of the extensor aspect of his left arm near the elbow of a 68-year-old man show a basal cell carcinoma associated with secondary localized cutaneous amyloid deposits presenting as a six by six millimeter flesh-colored nodule; the periphery of the nodule is outlined by purple lines (black arrow).

Cutaneous examination showed a six by six millimeter flesh-colored nodule on the extensor aspect of his left arm near the elbow (Figure 1). A shave biopsy of the superficial portion of the nodule was performed. The site was treated topically with mupirocin two percent ointment, three times daily, until it had healed.

**Figure 2:**
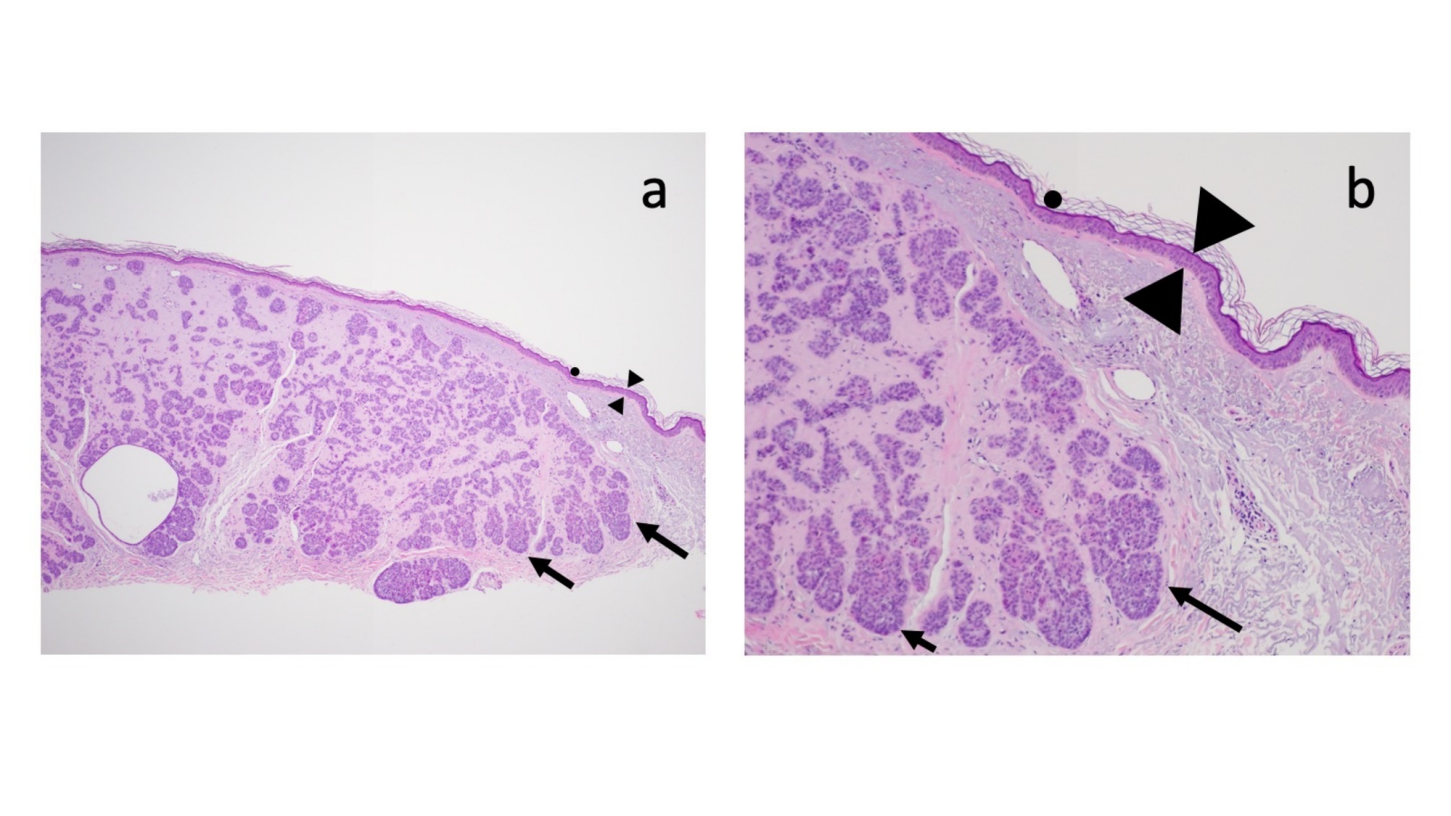
Microscopic features of basal cell carcinoma with amyloid deposits: dermal tumor. Distant (a) and closer (b) views of the biopsy specimen show sparse orthokeratosis (solid black circle) and a thin epidermis with flattening of the rete ridges (between the black triangles). Atypical basaloid tumor cells are present in the dermis as strands and nodular aggregates (black arrows) (hematoxylin and eosin: a, x 2; b, x 10).

**Figure 3:**
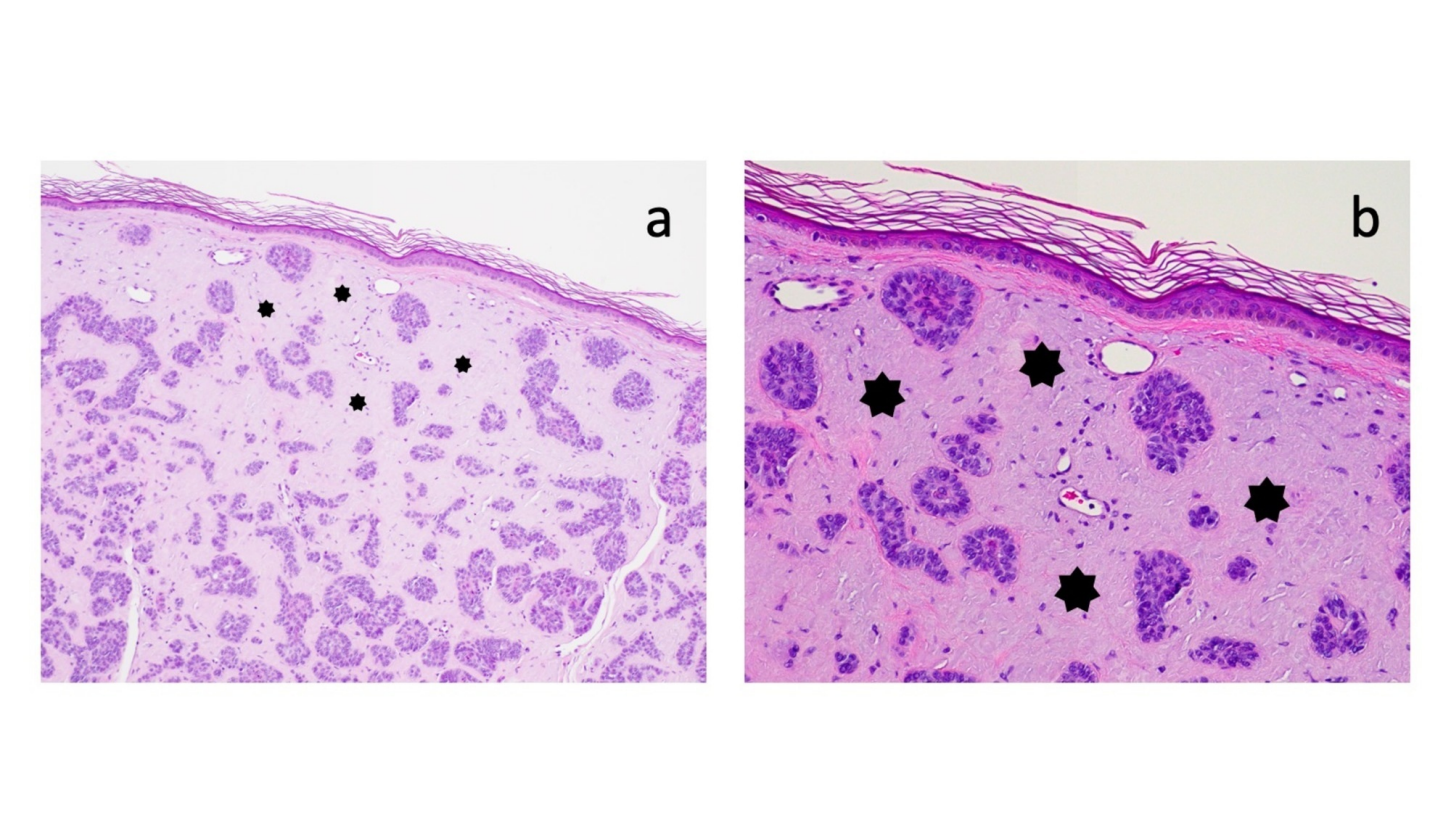
Microscopic features of basal cell carcinoma with amyloid deposits: amyloid. Distant (a) and closer (b) views of the biopsy specimen show marked deposition of amyloid, presenting amorphous material in the dermal stroma between the tumor aggregates (solid black stars) (hematoxylin and eosin: a, x 4; b, x 10).

**Figure 4:**
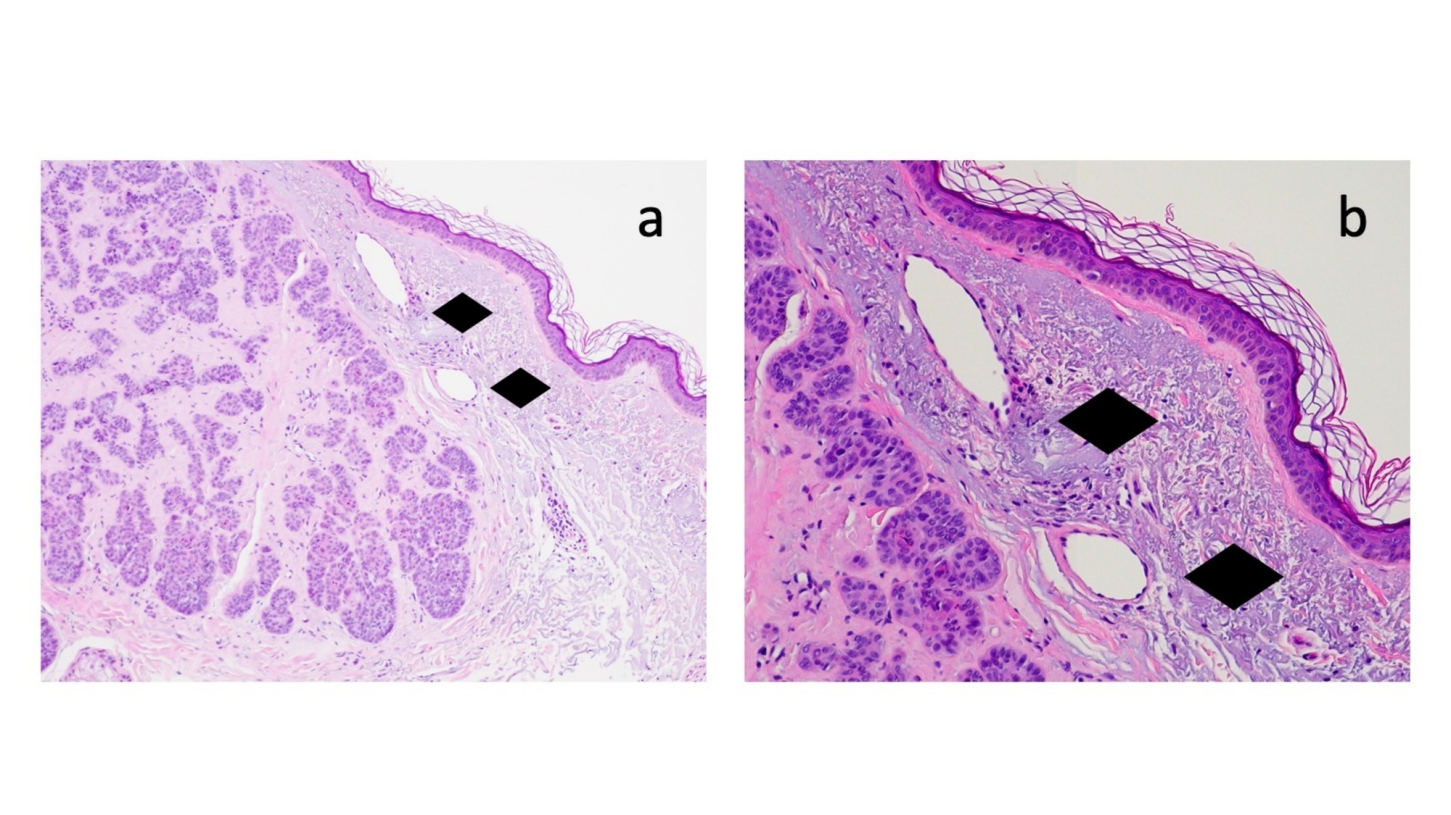
Microscopic features of basal cell carcinoma with amyloid deposits: solar elastosis. Distant (a) and closer (b) views of the biopsy specimen show solar elastosis presenting as homogenous alteration of the collagen in the dermis above the tumor and below the epidermis (solid black diamonds) (hematoxylin and eosin: a, x 10; b, x 20).

Microscopic examination of the hematoxylin and eosin-stained tissue specimen showed strands and nodular aggregates of atypical basaloid tumor cells in the dermis reaching the deep margin of biopsy; the overlying epidermis was thin with effacement of the rete ridges and sparse overlying orthokeratosis (Figure 2). Deposition of amorphous material filled the dermal stroma between the tumor aggregates (Figure 3); the amount of amyloid present was abundant (+++). The lateral aspect of the specimen showed solar elastosis in the upper dermis above the tumor and below the epidermis (Figure 4).

Correlation of the clinical presentation and pathological findings established the diagnosis of a nodular basal cell carcinoma with extensive amyloid deposition and adjacent solar elastosis. The residual tumor was excised and a side-to-side layered closure was used to resolve the surgical wound. The surgical site was healed without recurrence at a follow-up examination nine months later.

## Discussion

Basal cell carcinoma is the most common cutaneous malignancy. Clinical presentation includes various morphologies such as nodular, pigmented, red dot, sclerosing, and superficial. Several pathological variants of the tumor exist; microscopic examination permits the cancer to be differentiated—based on its biologic behavior—into aggressive subtypes (such as basosquamous, micronodular, sclerosing) and nonaggressive subtypes (such as adenoid, cystic, nodular, and superficial) [18].

Amyloidosis—characterized by the extracellular deposition of amyloid in tissue or organs—is localized or systemic, acquired or hereditary, and primary (AL type) or secondary (AA type). AL amyloidosis protein—from the deposition of immunoglobulin light chain fragments—occurs in patients with multiple myeloma and cutaneous nodular amyloidosis. In contrast, the deposition of AA amyloidosis protein—derived from keratinocytes—can be observed in patients with chronic inflammatory conditions and cutaneous lichenoid and macular amyloidosis [9, 19-20].

Secondary cutaneous AA amyloidosis can also occur in dermis accompanying benign and malignant tumors. Benign conditions and skin neoplasms, in addition to premalignant lesions, which have been associated with secondary amyloidosis include actinic keratosis, cylindroma, fibroma, melanocytic nevi, pilomatricoma, porokeratosis (disseminated superficial actinic and Mibelli), PUVA (psoralen and ultraviolet A)-treated skin, sebaceous gland hyperplasia, seborrheic keratosis, solar elastosis, and syringocystadenoma. In addition to basal cell carcinoma, secondary cutaneous amyloid deposition has been observed in the cancer lesions of patients with in situ and invasive squamous cell carcinoma, Merkel cell carcinoma, and mycosis fungoides [3, 7-9, 13, 15-16, 19].

Amyloid deposition in basal cell carcinoma has been most frequently described in retrospective pathology investigations (Table 1) [3-13]. These reports originate from many different continents. However, similar to the man in this report, individual patients with secondary cutaneous amyloidosis in their basal cell carcinomas have also been described from several nations including Germany [15], Korea [17], the UK [16], and the USA [14].

**Table 1:** Incidence of amyloid deposition in basal cell carcinomas.

Percentage of basal cell carcinomas with amyloid	Amyloid positive basal cell carcinomas	Total number of basal cell carcinomas	Nation of origin	Reference
74.6	194	260	Sweden	[3]
66.0	35	53	Malaysia	[4,5]
65.2	30	46	Australia	[6]
58.5	31	53	Taiwan	[7]
55.0	11	20	Turkey	[8]
50.8	101	199	UK	[9]
30.0	15	50	Turkey	[10]
17.2	5	29	Taiwan	[11]
12.8	22	172	India	[12]
11.4	10	88	USA	[13]

The percentage of basal cell carcinomas with secondary cutaneous amyloidosis in the English literature ranges from 11% to 75% (Table 1) [3-13]. The calculated incidence is 47% (454 of 970 basal cell carcinomas). Indeed, most of the retrospective studies of amyloid deposits in basal cell carcinomas (60%) observed an incidence between 51% and 75% (with a calculated incidence of 64%: 402 of 631 carcinomas) [3-9].

However, in the four studies the percentage only ranged from 11% to 30% (with a calculated incidence of 15%: 52 of 339 carcinomas) [11-13]. In addition, an earlier study in the non-English literature found the percentage to only be 8% (40 of 500 carcinomas) [9, 13]. The less frequent detection of amyloid deposits by these investigators may possibly be related to either the techniques used to detect amyloid [13] or the subtype of basal cell carcinoma that was evaluated [11] or both. Indeed, in one of the studies the researchers discovered depositions of amyloid in only 8% of the tumors (16 of 199 cases) based on their evaluation of hematoxylin and eosin-stained sections; however, the incidence of amyloid deposits increased to 51% (101 of 199 cases) when the same group of tumor specimens was examined after staining with methyl violet [9].

Deposition of amyloid in basal cell carcinomas was observed to occur more frequently in older patients [3]. In papers that noted 10 or less patients with amyloid deposits in their basal cell carcinomas, including the man in this report, the ages of the 22 individuals ranged from 47 to 84 years; the median was 67 years [11,13-17]. In a larger series of 194 patients whose carcinomas contained amyloid, more than half (106 individuals, 55%) were between 63 and 93 years of age [3].

Nodular basal cell carcinoma was the most frequent variant with amyloid deposits [3-5, 7-8]. However, deposition of amyloid was observed in various histological subtypes of basal cell carcinoma: adenoid [4-5, 9], cystic [4-5, 9], pigmented [7, 12], sclerosing [3-5, 9, 11], and superficial [4-5, 7-9]. In addition, tumors with mixed histology (sclerosing and either adenoid or nodular) were also observed [4-5, 9].     

Superficial basal cell carcinoma was the least common histological subtype observed [7, 9]. Indeed, two of the studies did not observe any tumors of this variant [4-5, 8]. However, one group of investigators found superficial basal cell carcinomas to be the second most frequent subtype [3].

Several researchers observed that amyloid deposits were more frequently observed in basal cell carcinomas with less aggressive histology patterns. Specifically, amyloid deposition was noted in nonaggressive variants of carcinoma in either 71% (25 of 35) [4-5], 73% (11 of 15) [10], or 90% (27 of 30) [6] of tumors; based on these studies, the calculated occurrence of amyloid deposits in nonaggressive basal cell carcinomas was 79% (63 of 80 tumors). The investigators postulate that the less aggressive basal cell carcinomas possess a higher apoptotic rate and, therefore, have a greater chance of amyloid formation [4-6, 10].

Amyloid deposition was initially identified—in most of the studies and reports—by retrospectively evaluating specimens that had been stained with hematoxylin and eosin [3-9, 12-13, 16-17]. Indeed, in several of the reports, the diagnosis of amyloid was based on the hematoxylin and eosin-stained sections. However, other sources of amorphous material in the dermis can mimic amyloid; in addition to solar elastosis (which was also present in the dermis of the reported patient), other etiologies for homogenous dermal depositions that may masquerade as amyloid include basement membrane material, colloid, and fibrin.

Additional examination for amyloid deposition, after processing the tissue with stains that allow amyloid to more readily be visualized, was also performed by some of the investigators; these stains included Congo red [3-6, 8-9, 12-14, 17], crystal violet [6-8, 13-14], methyl violet [9, 12], pagoda red [7], thioflavin S [6], thioflavin T [4-5, 13], and toluidine blue [13]. In some of the studies, either immunofluorescence stains or immunoperoxidase stains were performed to evaluate for immunoglobulins [6] or keratin [16], respectively. In addition, some of the researchers evaluated the tumor specimens with electron microscopy [4-6, 14-15].

The amount of amyloid present in the basal cell carcinoma was quantitatively measured in two studies on a scale ranging from + to +++ [3, 7]; a third investigation—in which 15 of the 50 tumors had amyloid deposits—also evaluated the amount of amyloid present but did not record the results [10]. One study, with 31 of 53 amyloid positive tumors, had nearly equal number of carcinomas with either + (10 of 31 tumors, 32%), ++ (nine of 31 tumors, 29%), or +++ (12 of 31 tumors, 39%) amount of amyloid deposits present [7]. The amount of amyloid deposition within the basal cell carcinomas significantly varied, in another study of 260 tumors with 194 amyloid positive tumors, from + (very sparse, 129 of 194 tumors, 67%) to ++ (47 of 194 tumors, 24%t) to +++ (abundant, 18 of 194 tumors, 9%) [3]. The amount of amyloid present in the reported patient was +++ (abundant). Indeed, the paucity of amyloid deposition in nearly two-thirds of the basal cell carcinomas with amyloid deposits that were observed in the larger study in which 260 tumors were evaluated may provide insight into a possible reason why some of the earlier studies [10-13] demonstrated a lower percentage of amyloid deposits in the basal cell carcinomas they reviewed.

Similar to the man in this report, the most common location of the amyloid deposits was in the stroma between the aggregates of basaloid tumor cells [3-5, 7, 9, 13]. Other dermal locations include either the papillary dermis above the tumor and in the immediate vicinity of the advancing front of the carcinoma [4-5, 9, 12-13]. Less commonly, deposition of amyloid was present within the tumor lobules [6,12-14] or at the outer edge of the upper portion of hair follicles up to one millimeter away from the tumor edge [9].

Amyloid positive basal cell carcinomas may be associated with concurrent solar elastosis. Again, similar to the reported patient, solar elastosis has been present in the dermis overlying or adjacent to the basal cell carcinoma [4-5, 12-13]. Indeed, in one study, 80% (28 of 35) of specimens that contained basal cell carcinomas with amyloid deposits also had solar elastosis [4-5].

The postulated pathogenesis for the development of amyloid deposits in the basal cell carcinomas has evolved. Initial researchers proposed that the dermal amyloid accompanying the basal cell carcinomas was produced by either falsely programmed fibroblasts [15] or fibroblasts that were influenced products released by the tumor cells [14]. Subsequent investigators attributed the amyloid deposition in the dermis to originate from degeneration and apoptosis of the basal cell carcinoma tumor cells [4-5, 12, 14]; indeed, one of these investigators observed that 74% of the amyloid positive tumors had ulceration and suggested that not only the presence of ulceration but also possible secondary infection and the long chronic duration of basal cell carcinomas resulted in the amyloid deposits [4-5].

However, the currently accepted etiology is that keratin from the cells in the overlying epidermis is the source of the amyloid deposition in basal cell carcinomas. Apoptosis and damage to the keratinocytes in the epidermis occur. Subsequently, after filamentous degeneration of these epithelial cells, secondary amyloid AA protein forms in the dermis [7-9, 16].

The deposition of amyloid does not alter the management of basal cell carcinomas. Mohs microscopic surgery may be utilized for tumors that have aggressive pathology subtypes, are located on the face and other sites prone to recurrence, or both. For nonaggressive tumors located at other areas, various treatment options—some of which include excision, curettage and desiccation and topical imiquimod cream or fluorouracil cream—exist [18].

## Conclusions

Amyloid deposition may be present in basal cell carcinomas. However, the observed incidence of this phenomenon has varied from 11% to 75%. The lower detection of amyloid observed by some of the investigators may result from staining technique or tumor subtype or quantity of amyloid present; indeed, a significant increase in the number of randomly selected basal cell carcinomas containing amyloid after staining with methyl violet (51%) was observed as compared to the original evaluation of hematoxylin and eosin-stained specimens (8%) by one group of researchers. Basal cell carcinoma with amyloid deposition was discovered more frequently in older individuals with less aggressive tumor histology subtypes. The most common tumor with amyloid deposits was nodular basal cell carcinoma; the least frequent, superficial basal cell carcinoma. The amyloid deposits were usually identified using hematoxylin and eosin and subsequently confirmed using stains that allowed for easier visualization of the amorphous material. The amyloid deposits were most commonly located in the stroma between the tumor aggregates; the amyloid could also be found within the aggregates of carcinoma or in the dermis either above or at the advancing edge of the basal cell carcinoma. In addition, similar to the reported patient and in up to 80% of specimens in one of the studies, many of the basal cell carcinomas with amyloid deposits also contained solar elastosis. Secondary amyloid AA protein, from keratin derived from the epithelial cells overlying the basal cell carcinomas, accounts for the etiology of the amyloid deposition in these tumors. The management of amyloid containing basal cell carcinomas is not altered by the presence of the amorphous material; indeed, the treatment of the tumor is the same as when the basal cell carcinoma does not contain amyloid deposition.
